# What is a vector?

**DOI:** 10.1098/rstb.2016.0085

**Published:** 2017-03-13

**Authors:** Anthony James Wilson, Eric René Morgan, Mark Booth, Rachel Norman, Sarah Elizabeth Perkins, Heidi Christine Hauffe, Nicole Mideo, Janis Antonovics, Hamish McCallum, Andy Fenton

**Affiliations:** 1 Vector-borne Viral Diseases Programme, The Pirbright Institute, Ash Road, Pirbright, Woking GU24 0NF, UK; 2 School of Veterinary Sciences, University of Bristol, Life Sciences Building, 24 Tyndall Avenue, Bristol BS8 1TQ, UK; 3 School of Medicine, Pharmacy and Health, Durham University, Thornaby TS17 6BH, UK; 4 School of Natural Sciences, University of Stirling, Stirling FK9 4LA, UK; 5 School of Biosciences, Cardiff University, Sir Martin Evans Building, Museum Avenue, Cardiff CF10 3AX, UK; 6 Department of Biodiversity and Molecular Ecology, Centre for Research and Innovation, Fondazione Edmund Mach, Via E. Mach 1, 38010 S Michele all'Adige (TN), Italy; 7 Department of Ecology and Evolutionary Biology, University of Toronto, Toronto, Ontario, Canada M5S 3B2; 8 Department of Biology, University of Virginia, Charlottesville, VA 22904, USA; 9 Environmental Futures Research Institute, Griffith University, Nathan 4111, Queensland, Australia; 10 Institute of Integrative Biology, University of Liverpool, Liverpool L69 7ZB, UK

**Keywords:** vector, transmission, arbovirus, disease ecology, host–pathogen interactions, public health

## Abstract

Many important and rapidly emerging pathogens of humans, livestock and wildlife are ‘vector-borne’. However, the term ‘vector’ has been applied to diverse agents in a broad range of epidemiological systems. In this perspective, we briefly review some common definitions, identify the strengths and weaknesses of each and consider the functional differences between vectors and other hosts from a range of ecological, evolutionary and public health perspectives. We then consider how the use of designations can afford insights into our understanding of epidemiological and evolutionary processes that are not otherwise apparent. We conclude that from a medical and veterinary perspective, a combination of the ‘haematophagous arthropod’ and ‘mobility’ definitions is most useful because it offers important insights into contact structure and control and emphasizes the opportunities for pathogen shifts among taxonomically similar species with similar feeding modes and internal environments. From a population dynamics and evolutionary perspective, we suggest that a combination of the ‘micropredator’ and ‘sequential’ definition is most appropriate because it captures the key aspects of transmission biology and fitness consequences for the pathogen and vector itself. However, we explicitly recognize that the value of a definition always depends on the research question under study.

This article is part of the themed issue ‘Opening the black box: re-examining the ecology and evolution of parasite transmission’.

## 1. Introduction

Many parasites and pathogens responsible for some of the most important diseases in humans, agriculture and nature are routinely described as ‘vector-borne’. These include emerging parasites and pathogens such as dengue virus throughout the tropical world [1], West Nile virus in North America [2] and Europe [3], Crimean–Congo haemorrhagic fever virus in Turkey [4], hantavirus in Europe [5], bluetongue virus in Europe [6], zika virus in South America [7], Lyme borreliosis in Europe [8] and chikungunya virus in the Caribbean [9]. Almost 20% of human deaths are caused by infectious diseases that are described as vector-borne, chiefly malaria, yellow fever, leishmaniosis, trypanosomiasis, Chagas' disease and Japanese encephalitis [10], and such diseases are predicted to present a growing threat in the near future [11]. However, different definitions of a vector are used in different fields. For instance, the term is universally applied to haematophagous arthropods, such as *Ixodes* ticks that transmit *Borrelia burgdorferi* or *Aedes* mosquitoes that transmit dengue virus, but the term ‘vector’ has also been applied to badgers transmitting *Mycobacterium bovis* [12–14], dogs transmitting rabies virus [15], snails transmitting *Schistosoma* flatworms [16,17] and rodents transmitting hantaviruses [18]. Clearly a large number of definitions of ‘vector’ are currently being used, and the question in any multi-host system should be to ask when and why a particular host in that system warrants designation as a ‘vector’.

This is perhaps most easily understood by considering the simplest canonical case, namely a one pathogen, two host species system. If the pathogen is present in each of the two species of hosts, and transmission between those species is required to maintain the pathogen in the system, there is no inherent theoretical reason why one or other species should have the designation of ‘host’ or ‘vector’. In principle, a full understanding of the dynamics of the system requires knowledge of the contributions and feedbacks involving all participants, and the outcome will be independent of what designations are given to them. Nevertheless, the designation of one or the other host as a vector is commonplace in the literature on infectious diseases. It is therefore of interest to explore the factors that have gone into defining one or other species as a vector, why such a distinction has proved useful, and conversely, if there are dangers involved in pursuing these definitions.

**Figure 1.**
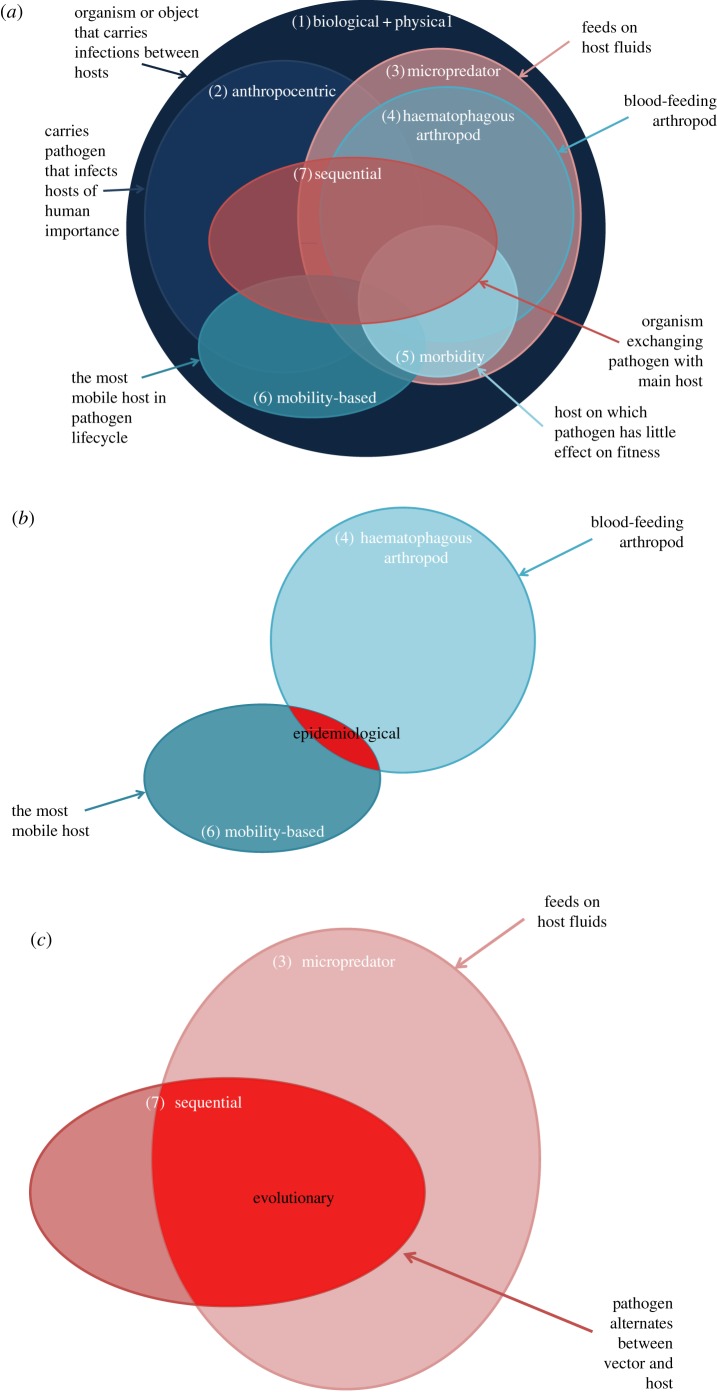
(*a*) Schematic of the relationship between the various vector definitions provided in §1. (*b*,*c*) Suggested definitions from the epidemiological and evolutionarily perspectives respectively.

We first review some of the most common uses of the term (summarized in figure 1*a* and table 1), a number of which we immediately dismiss, either because we believe they are too broad or too narrow to be of practical use. We then consider in more detail which definitions are most appropriate for different contexts, and which aspects of host–pathogen–vector biology are most important when considering the most appropriate definition of a vector.

**Table 1.** How some potential ‘vectors’ map onto the definitions discussed in the text. ✓, true; 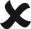, false; ?, unclear or debatable (not counted in totals).

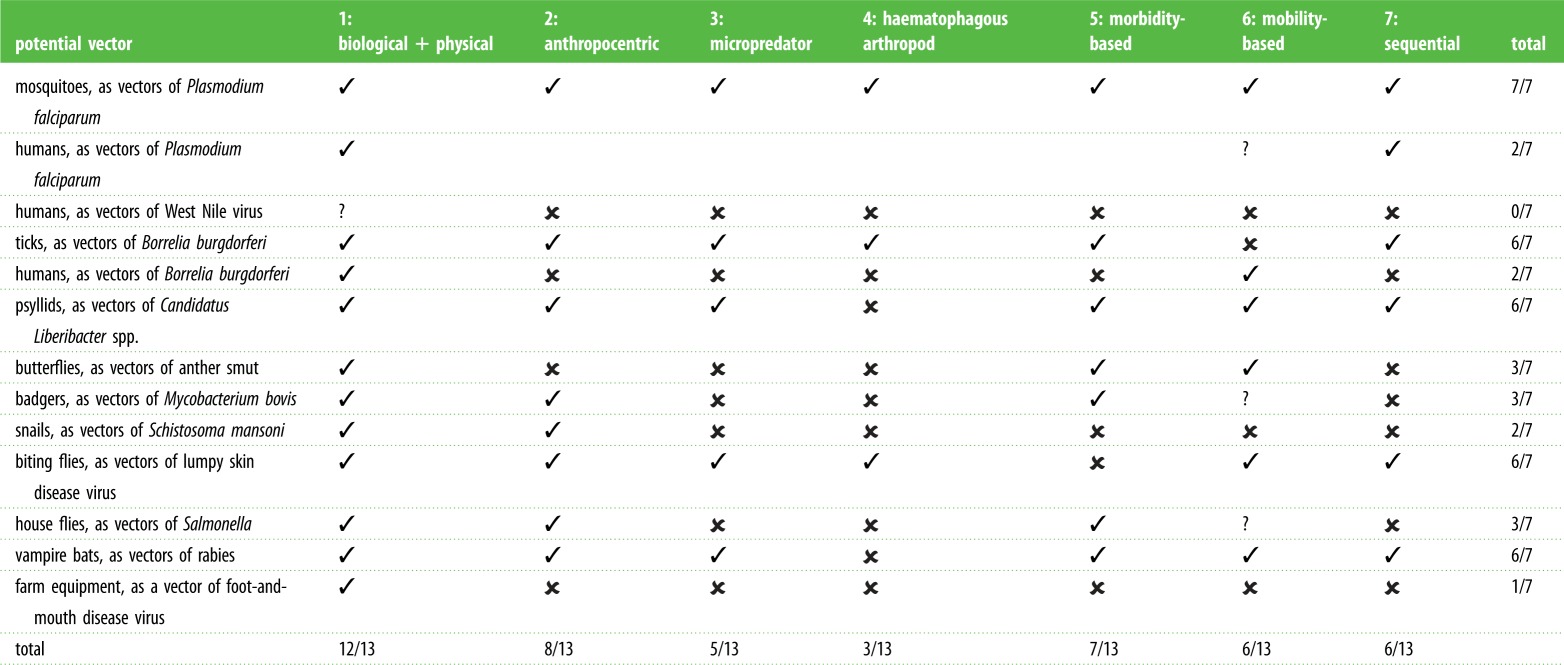

## 2. An overview of existing definitions of ‘vector’

One of the broadest definitions defines a vector as any organism (vertebrate or invertebrate) that functions as a carrier of an infectious agent between organisms of a different species [19]. This includes organisms playing a purely mechanical role in transmission (e.g. *Musca* flies in the transmission of *Chlamydia trachomatis*, the causative agent of trachoma). Some authors have gone further and extended the definition to include fomites (the biological + physical definition; definition #1)—inanimate objects capable of carrying infectious material and transferring it between hosts, such as syringes [20] and paper money [21]. Although it seems incongruous to group fomites together with biological agents of transmission, which can experience strong ecological and evolutionary interactions with the pathogen, from a public health perspective this definition may be relevant to disease management and prevention. Alternatively, a relatively common way to assign vector status to a particular host in a multi-host system is with reference to their involvement in the transmission of pathogens of human relevance (*anthropocentric*, definition #2). These may be pathogens that directly infect humans, for example, ‘[v]ectors are living organisms that can transmit infectious diseases between humans or from animals to humans' [22] (and [23], with slightly different wording); under this definition, any non-human host connected to human hosts by one or more transmission modes is a vector. While the motivation behind such a definition seems obvious, it clearly has problems if applied rigidly; for example, it leads us to the slightly illogical consequence that under this definition a mosquito transmitting West Nile virus (WNV) from a wild bird to a human is a vector, while a mosquito transmitting WNV between wild birds is not. A slightly more flexible interpretation would be that any host capable of transmitting a pathogen of importance to humans to or between one or more hosts is considered to be a vector.

One of the most obvious definitions is based on the recognition that most organisms we commonly recognize as being ‘vectors’ are hosts that transmit a pathogen while feeding non-lethally upon the internal fluids of another host. Largely this definition overlaps with the *micropredator* classification proposed by Lafferty & Kuris [24, p. 509], defined as ‘a natural enemy [that] attacks more than one victim…and does not necessarily eliminate its fitness’ (definition #3). This definition covers many key points fundamental to vector biology: contact (feeding) occurs more than once during a micropredator's lifetime (otherwise, it has no opportunity to transmit a pathogen between hosts) and contact improves the fitness of the feeding vector (micropredator) while reducing the fitness of the ‘other’ host (although perhaps negligibly) to a value greater than zero. One advantage of this definition is that it clearly differentiates a vector from an intermediate host (such as *Biomphalaria* water snails within the *Schistosoma* transmission cycle), where definitive and intermediate host fitnesses are not directly affected by each other.

A related definition is the *haematophagous arthropod* definition (definition #4), which defines vectors only as blood-feeding arthropods such as mosquitoes, ticks, sandflies, tsetse flies and biting midges [25]. Such arthropods generally also fall within the micropredator definition above, with the exception of species that feed on only a single host in their entire lifetime, such as louse flies (Hippoboscidae) and one-host ticks (such as *Rhipicephalus microplus*). This definition is used explicitly by several groups including the European Centre for Disease Prevention and Control [26] and the Intergovernmental Panel on Climate Change [27], and other sources either implicitly adopt this definition [28,29] or explicitly cite a broader definition but go on to discuss only examples falling under this definition [30]. A weakness with such a definition is that it may detract attention from useful insights from species playing essentially equivalent roles in non-vertebrate hosts, for example sap-feeders (aphids) or haemolymph feeders (*Varroa* mites). In addition, other large groups of vertebrates, such as rodents, which also spread pathogens through their saliva (or other excreta, albeit generally by a different route from percutaneous penetration), and that are often considered vectors, are also excluded.

An alternative perspective for defining vectors is one that emphasizes some functional aspect of the vector's life history, or that of its interaction with the pathogen. For example, the *morbidity-based* definition (definition #5) describes a vector as a host within a multi-host transmission cycle for which infection does not significantly reduce that host's fitness. However, while fitness effects of the pathogen on organisms universally accepted as vectors are often not overt, they have been frequently observed experimentally: for example, effects on fecundity [31], feeding frequency [32] or feeding duration [33]. Alternatively, the *mobility-based* definition (definition #6) defines vectors as the most mobile host in a transmission cycle of two or more hosts. This definition frames the distinction in terms of parameters likely to have consequences for epidemiology (in this case, typical spatio-temporal patterns of spread), offers the advantage of simplicity and fits most disease systems traditionally considered to be vector-borne. However, under this definition, ticks would not be defined as vectors because they are typically less motile than their host. In addition, ‘vector’ identification using this definition may be difficult in practice; for example, insect vectors may occasionally be blown very long distances under certain atmospheric conditions [34] but their typical lifetime dispersal distance will be shorter than that of many avian hosts. Given that there are obvious species that we would intuitively regard as being vectors that are excluded by these last two definitions, we suggest both the morbidity- and mobility-based definitions by themselves are neither sufficient nor necessary to describe a vector.

Some differences in applicability between each of these definitions are illustrated in table 1 and their relationships with each other are illustrated in figure 1*a*. Clearly, each definition emphasizes different aspects of vector–pathogen-definitive host biology, but there may also be substantial overlaps between them. When, then, is ‘vector’ a useful definition, and under what contexts are different definitions applicable? In what follows, we consider from a variety of perspectives which definitions are most useful, and the key aspects of host–vector–pathogen biology that need to be captured within any meaningful definition of ‘vector’.

## 3. What definition of ‘vector’ is useful for understanding pathogen transmission: is a vector different from other hosts?

A vector could be considered just another host in a parasite's life cycle, and applying some of the above definitions to multi-host systems can result in the classification of two or more different groups as ‘vectors', implying that it is appropriate to use similar ways to represent them in mathematical models (as also discussed in [35]). Here, we discuss when this is a sensible simplification and also when it may obscure or conceal important epidemiological and ecological processes.

### (a) The population dynamics perspective

Multi-host–pathogen systems are often described theoretically within the framework of next-generation matrices [36,37] or multi-species dynamic models [38,39]. These theoretical frameworks provide a very clear distinction between ‘vectors’ and other host species within a multi-host context, based on how those hosts contribute to the pathogen's basic reproduction ratio (*R*_0_). *R*_0_ is the expected number of new infections generated by a single infected individual in a wholly susceptible host population (or multi-host community), and so represents the potential for the pathogen to invade a naïve community, but also under some conditions it can be used to describe the contribution different hosts make to endemic persistence [40] or pathogen evolution [41; see §4b]. In the case of a pathogen circulating within a community of multiple ‘equivalent’ host species, where transmission may occur within and between species, the pathogen's overall *R*_0_ is given by an expression of the form (shown here for two host species, one of which is a putative vector):
3.1
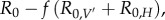
where the overall *R*_0_ is proportional to the sum of the reproduction ratios in hosts and putative vectors (denoted by the subscripts *H* and *V*′, respectively). Importantly, however, with a ‘true’ vector, pathogen transmission occurs through sequential, and repeated, feeding of the vector on the ‘other’ host species, which gives rise to an alternative *R*_0_ expression of the form:
3.2
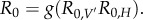


Now the overall reproductive ratio is proportional to the product of the reproduction ratios in the host and putative vector. Equation (3.1) most closely captures the biology of ‘multi-host’ models, where pathogens have multiple potential transmission routes among hosts, i.e. there may be transmission between multiple host species, but infection of either can be independent of the other [39–41]. A key point here is that the different host species are to an extent ‘substitutable’ in equation (3.1) [42], and therefore their combined contributions to pathogen fitness are additive. Conversely, the biology implicit in expression (3.2) is fundamentally different, as pathogens are now constrained to infect a host and vector sequentially. This form of *R_0_* is characteristic of many theoretical models of vector-borne transmission [41,43,44], whereby pathogen fitness is defined as the average number of new infected vectors produced by a single infected host, multiplied by the expected number of new infected hosts generated by each of those vectors, again reflecting the sequential passage through vector and host. Therefore, from a pure population-dynamic theory point of view, a vector–host system can be distinguished from other multi-host systems by this multiplicative form of the pathogen's basic reproductive ratio (*sequential*; definition #7).

Importantly, this distinction arises purely from consideration of the population dynamics of pathogen transmission. As such, it overlooks other aspects of vector–host–pathogen biology that may be relevant in different contexts. For example, a definition purely based on the functional form of the *R*_0_ relationship (equation (3.2)) would rule in many so-called ‘intermediate’ hosts (e.g. snails as hosts for schistosomes) as vectors, if they are an obligatory (sequential) host in the pathogen's life cycle. Because they play different roles in parasite life cycles, it seems appropriate that these different host types (vectors, which transmit a parasite or pathogen, and intermediate hosts, which are necessary for a parasite to complete its life cycle) should not necessarily be grouped under the same umbrella term. To separate those host types it may therefore be necessary to refine this definition, for example to include aspects of the ‘micropredator’ definition to emphasize the feeding component, and direct contact of the vector with the host, typical of the majority of considered vector species (figure 1*c*). In what follows, we consider additional/alternative aspects of vector–host–pathogen biology that may influence our definition of vectors.

### (b) Timescales and lifespan

Timescales are a critical consideration. If there are hosts that move, reproduce and die much more quickly than the other hosts in the system, then it may be useful to consider them separately from other hosts. A standard practice in simplifying complex models of host–parasite systems is to assume that short-lived life-history stages are at ‘quasi-equilibrium’ with the current population sizes of the longer-lived life-history stages [45]. This reduces the dimensionality of the problem for modelling and data gathering. There will be other circumstances in which the most parsimonious way of understanding pathogen transmission, spread and management is to use an expression such as ‘vectorial capacity’, which subsumes the within-host processes that occur within the vector, and the vector population dynamics, into a single expression [46]. Transmission between susceptible and infected hosts is assumed to occur at a rate dependent on the characteristics of the vector and host populations at that particular time, without considering as important dynamical changes in either the vector population or the prevalence of infection in the vectors that might occur in the time between vectors acquiring infection and transmitting it to a further host. However, these simplifications are not helpful for describing the behaviour and epidemiological role of hard ticks such as *Ixodes ricinus*, which transmits *B. burgdorferi* and tick-borne encephalitis virus (TBEV) [35]. These ticks typically live for several years, longer than many of their vertebrate hosts [37], and the intervals between the single feeding of each life stage may be up to a year. While ticks are often described as ‘vectors’, the structure of the models necessitated by the substantial differences in lifespan, feeding and mobility between ticks and their hosts means that most of the simplifications that are commonly assumed for ‘vectors’ are not appropriate and they are essentially modelled as another host [47].

Where transmission between different host species funnels through one or a small number of species, then recognizing these differences via a special designation (whether ‘vectors’ or another term) may be helpful. In the case of *B. burgdorferi* and TBEV, in many ecosystems, one species of *Ixodes* tick acts as a nexus transferring infection between a large number of mammalian host species [48]. Here the important point is that the vectors (ticks in this case) are sequential hosts in the pathogen's life cycle (matching our definition #7), and this single category of hosts therefore represents a particularly vulnerable target to interrupt transmission and manage the risk of spillover to humans. Applying this ‘nexus’ definition of a vector would, however, lead to some hosts generally not considered as vectors being classified as such. For example, *Toxoplasma gondii* infects a very wide variety of mammalian hosts, but continued transmission requires a felid definitive host [49].

### (c) Frequency-dependent versus density-dependent contact

In terms of classical approaches to modelling infectious diseases, a key component of many models of vector-borne infections is the assumption of frequency-dependent (FD) transmission, as distinct from density-dependent (DD) transmission. In the case of DD transmission, the rate at which an individual contacts other individuals depends on the density of infected individuals; as a consequence, as density increases, transmission rate will increase [50]. On the other hand, for frequency-dependent transmission, it is assumed that an individual has a fixed number of contacts per unit time that is independent of the population size, and so the rate of transmission depends on the frequency (proportion) of infection among those contacts [50].

The dynamics of transmission are very different for these two cases, and certain modes of transmission are more appropriately modelled as one or the other; for example, transmission via droplet or aerosol is density-dependent (high host densities result in more rapid spread), whereas the rate of infective contact via sexual transmission is not. It may be possible to predict the nature of the transmission function for a known system with a reasonable understanding of the biology of the organisms involved [51]; for example, sexual transmission may be largely frequency-dependent, as most individuals have a constant number of sexual contacts per unit time, regardless of population density. For some groups typically identified as vectors, such as mosquitoes, frequency-dependent transmission is likely to be the most appropriate; females need to feed every few days, for which they will actively seek a host and although the density of hosts may make that more or less easy, they are likely to be able to find a host even at low density. On the other hand, many tick species are relatively immobile and rely on hosts brushing past them. If the density of hosts increases, then the ticks are more likely to find a host. In this case, density-dependent transmission is more appropriate.

Clearly, from the perspective of a mathematical epidemiologist, it is not particularly helpful to have a definition of vector that encompasses hosts which exhibit both density- and frequency-dependent rates of potentially infectious contact, because they must be represented differently within modelling frameworks. Furthermore, a definition of ‘vector’ that suggests that HIV is vector-borne but *B. burgdorferi* is not is unlikely to satisfy most people. The relationship between population density and transmission is therefore likely to be acceptable as a qualification for defining a vector only in combination with other traits.

A perhaps more basic problem with using this definition is that it assumes that contact rate functions can be strictly classified as one or the other. In practice, many attempts to characterize natural populations within this paradigm have found results intermediate between these two extremes, and it may be more helpful to think of this distinction as a spectrum rather than a dichotomy [52]. Hence, it seems unlikely that the functional form of transmission from population modelling (i.e. frequency-dependence versus density-dependence versus an intermediate) provides a sufficient means of classifying vectors.

### (d) Usefulness of definitions for control

Defining a class of hosts as a ‘vector’ or otherwise differentiating them on certain criteria may help in predicting patterns of spread or the likely effectiveness of certain control strategies. Here, the ecological definitions (particularly the ‘haematophagous arthropod’ definition 4) are most likely to be useful, as many groups of haematophagous arthropods share characteristics with clear consequences for epidemiology or control, including ectothermy (as a result of which pathogen replication within the vector and some key biological functions such as the rate of blood-feeding or egg production are more strongly linked with environmental temperature), a relatively short lifespan and high intrinsic rate of reproduction (as a result of which population sizes can be affected by short-term environmental change). They may also possess ecological and metabolic similarities such as aquatic juvenile stages (rendering them susceptible to control strategies such as the removal or treatment of ephemeral water bodies), flight, or vulnerability to similar control products such as certain chemicals (e.g. neonicotinoids) or bacteria (e.g. *Bacillus thuringiensis*). At the same time, overly broad definitions will not be helpful; most strategies effective at interrupting the spread of malaria or dengue will not be applicable to the control of schistosomiasis or rabies.

### (e) Insights from applying vector status to unusual systems

Leaving definitions aside, vector-borne disease theory might be usefully applied to hosts or objects not usually considered as such. Parasitic helminths are responsible for transmitting several economically important pathogens in plants [53]. The strategies adopted by helminths to find their host could be also exploited to enhance pathogen transmission between vertebrates, in the same way as for arthropod vectors (see [54] for a review). For example, the protozoan cause of blackhead disease in turkeys, *Histomonas meleagridis*, is transferred to the egg of the caecal nematode *Heterakis gallinae*, and passed onwards to birds by the ingestion and subsequent hatching of larvated worm eggs [55]. Whereas earthworms can act as transport hosts of *Heterakis* and in that way transfer *Histomonas* [56], the role of the nematode is essential for transmission, and functionally it might be considered a vector. The role of helminths as disease vectors has been little examined in spite of examples of pathogen carriage by helminths, especially in plant pathology. In some cases, synergies and co-pathologies occur when both are co-located in a host. Given that vectoring results in co-infection, this situation is likely to be common. *Wolbachia* endo-bacteria in filarial nematodes, for example, appear to be responsible for aspects of filarial disease [57], whereas the trematode *Fasciola hepatica* modulates host immunity and increases the establishment and persistence of bacteria such as *Salmonella* [58] and *Bordetella* [59].

A combined micro- and macro-parasite modelling framework has been used to investigate the potential vectoring of bacteria by parasitic nematodes [54]. Results showed that coexistence of vectored and directly transmitted phenotypes within pathogen species was likely across a range of parameters, even when vector efficiency was high, and that long survival of free-living helminth stages could offset high mortality in the definitive host and enable the persistence of virulent pathogens. High degrees of helminth aggregation made vectoring less beneficial for the pathogen through increased helminth-induced host mortality, in contrast to arthropod-borne vectors, in which direct costs of ectoparasitism are rarely accounted for and aggregation can increase vector efficiency through co-feeding [60]. This example shows that viewing a novel disease system as vector-borne can help to predict how that system might behave in nature, and assess the plausibility of vectored and other transmission routes. Contrasts in predicted and observed behaviour between pathogens vectored by novel/putative, and more traditional, vectors can lead to better understanding of what drives behaviour across a range of vector-borne disease systems. Empirical work further explored the potential for parasitic helminths to harbour bacteria [61], and using a tractable system (non-parasitic, free-living helminths) asked what advantages might be conferred to pathogens that are associated with helminths. *Salmonella* bacteria were found to survive adverse environmental conditions better when within the free-living nematode *Caenorhabditis elegans* [62]. This included ultraviolet light and low pH, such that carriage within nematodes could both provide an environmental reservoir of infection for food-borne bacteria and protection against host defences such as stomach acid. Given the fact that polymorphism in transmission strategy could arise in such a system [54], this raises the question of when facilitation of transmission such as this becomes vectoring.

The study of vector-borne disease has provided theoretical frameworks and insights that can be applied usefully to other systems. Hypodermic needles, for example, might be considered as vectors under definition #1, and pseudo-biological characteristics defined, such as rates of birth (entry of new needles into the population), infection (contamination) and death (removal or needle exchange), whereas the use and reuse of needles is analogous to biting rate. This thought model has been applied to the problem of HIV transmission and supported needle exchange as part of harm reduction approaches to disease control [20]. Thus, decreasing proportions of needles positive for pro-viral DNA fell as increasing cumulative numbers of clean needles were provided, as a result of decreasing circulation time, an effect equivalent to that of decreasing vector survival rate [63]. In this case, therefore, considering inanimate objects as vectors was useful, whatever the legitimacy of that definition. Creative use of vector theory should, perhaps, not be constrained too strictly by ontology.

## 4. What definition of ‘vector’ is useful for understanding parasite and pathogen evolution?

### (a) Defining vectors based on contributions to pathogen fitness

As described above, theoretical studies of multi-host systems often seek to characterize the functional form of different host species’ contributions to the basic reproduction number, *R*_0_, of the pathogen. Although primarily an ecological measure of the pathogen's ability to invade a naive host community, it can also be used in an evolutionary context as an operational definition of pathogen fitness [64,65]. Given a mathematical expression for *R*_0_, such as those presented above, one can ask how changing a pathogen trait of interest alters *R*_0_; hence, one can predict the evolutionary trajectories of those traits under different selection scenarios and trade-offs. In particular, from an evolutionary perspective we suggest that it is important to recognize that the key defining feature of vector transmission is that every pathogen generation (i.e. passing from one infected host to another infected host) involves contact with the vector [41]. As such, there is clear overlap with the ‘sequential’ definition of a vector from population dynamics theory. However, additional considerations are also relevant from an evolutionary perspective. For example, if we assume that a vector-borne pathogen is typically transmitted through feeding by the vector, then the evolutionary interests of the pathogen may be expected to at least partially align with that of a vector. However, if the pathogen was instead transmitted trophically (e.g. through consumption of an intermediate host by a definitive host), then the evolutionary interests of pathogen and intermediate host would conflict [66] (though not if the intermediate host is itself a parasite—see above). Such conflicting selection pressures are seen in the evolution of host manipulation strategies by trophically transmitted parasites, which increase the likelihood of an infected intermediate host being predated by the parasite's definitive host [67]. Hence, although such trophically transmitted parasites and ‘true’ vector-borne parasites would have *R_0_* expressions of the same functional form (e.g. equation (3.2)), they would have very different evolutionary dynamics; this further emphasizes the need to differentiate vectors and intermediate hosts.

### (b) How much vector biology should be included in models of pathogen evolution?

A clear and relevant vector definition is potentially very helpful in offering insights into pathogen evolution, as it can illuminate key aspects of epidemiological systems that are critical for pathogen evolutionary processes. Despite this, many evolutionary and ecological models simplify or ignore much of the complexity of vectors. Like in the ecological models discussed in §4a, vectors are often treated as mobile syringes rather than organisms in their own right, and their broader ecology and behaviour are frequently subsumed into a black box described by their biting and mortality rates. Subsuming vector biology into a few vital rates of only the vector is analogous to subsuming or ignoring the mechanistic details of within-host dynamics, and only dealing with among-host processes (as in classical epidemiological models) for studying pathogen evolution: in both cases, there is no opportunity for reciprocal feedback from the simplified level (within-hosts or within-vectors) to the between-host level [68]. For vector-borne diseases, there will be reciprocal feedback when a pathogen trait that is important in a focal host also influences interactions within the vector (e.g. through immune stimulation), or alters vector feeding behaviour, or impacts vector mortality or fecundity.

As an example, one pathogen trait for which interactions in the host and vector are likely to be influencing pathogen evolution is the production of transmission stages by malaria parasites. Because one infected red blood cell in a vertebrate host can produce multiple asexual parasites (capable only of infecting other red blood cells) or one transmissible parasite (required for infecting a mosquito vector), the proportion of infected cells that produce the transmissible stages is a ‘trait’ that is expressed in a host. Because, all else being equal, the more transmissible stages are produced in a given cohort of infected cells, the fewer red blood cell-destroying asexual parasites are produced, it is also a trait with clinical significance. A few theoretical studies have explored the evolution of this trait [69–75] but invariably have included no mechanistic description of within-vector interactions. However, inside a vector, these transmissible stages fuse, form oocysts, and eventually release motile parasite stages that can be transmitted to another vertebrate host. Experimental data suggest that the density of gametocyte stages that make it into a vector may be inversely related to the density of stages that are available to be transmitted out of the vector [76], thus influencing the probability of transmission through a vector bite. This is clearly a case where interactions in the vector—an essential, sequential host in the parasite's life cycle—are influencing the evolution of a trait expressed in a definitive host.

Intuition might suggest that a trait like the production of transmissible stages would influence transmission to vectors and performance in vectors, but for some other traits of interest it might not be so clear if interactions in the vector will modify or constrain evolution. Unexpected genetic correlations may invisibly influence the evolution of important pathogen traits. Malaria parasites, for example, are evolving resistance to current front line antimalarial drugs, and the putative mutations responsible appear to be in close proximity to a gene that is associated with evasion of mosquito immunity [77–80], leading to the interesting speculation that mosquito–malaria interactions may constrain the evolution of drug resistance [81], or that the evolution of drug resistance may alter the suite of mosquitoes that are able to transmit drug resistant strains [82]. As experimental and genetic data continue to shed light on within-vector interactions that might influence pathogen evolution, more of this biology ought to be built into evolutionary models.

### (c) Dead end or partial vectors

A high proportion of individuals within a population that are exposed to a potentially infectious dose of a pathogen may fail to develop a fully disseminated, transmissible dose under ‘typical’ infection conditions [83,84]. Similarly, some species may be capable of developing disseminated infections with a pathogen but rarely or never encounter it under natural conditions and/or are unable to transmit it to other hosts [85]. Such ‘dead end’ hosts may be considered important as indicators of the distribution of a disease or for reasons of public or animal health, such as human and equine cases of West Nile virus infection, neither of which attain transmissible levels of viraemia. In contrast, ‘dead end’ vectors are rarely considered as they are typically assumed to be of little or no epidemiological importance. However, from an evolutionary perspective, they may offer important insights into how the vector-borne transmission mode originally evolved for a pathogen (see also [86]), or help to identify the potential for a pathogen to shift to novel transmission routes or hosts in the future.

## 5. What is the best definition?

As we have seen above, and summarized in figure 1, multiple definitions of vector are in common use. We suggest that the broadest definitions (e.g. the biological + physical definition) de-emphasize potentially critical differences between superficially similar vectors, for example insects and ticks [35], or encourage over-simplification of the interactions between vectors and pathogens. Conversely, some other definitions (e.g. anthropocentric) are too narrow and/or subjective to be of practical use, excluding many species that would intuitively be regarded as being vectors (e.g. just because they do not feed on humans). However, it is critical to recognize that using any single definition carries the risk of over-simplification, and there may be different appropriate definitions depending on the context. For example, it is often of practical benefit when studying certain systems (e.g. transmission networks of two or more host groups in which one host, essential to the life cycle, is a flying blood-feeding insect within which pathogen replication occurs) to highlight similarities between such hosts. Benefits to recognizing commonalities among these species include similarities in metabolism and response to environmental change, ecology and breeding site preferences, the nature of and spatio-temporal patterns of contact with other hosts (owing to feeding behaviour, mobility, etc.) and similarities in vulnerability to certain control strategies. As such, the intersection of the ‘haematophagous arthropod’ (#4) and ‘mobility’ (#6) definitions are the most useful from a medical and veterinary perspective (figure 1*b*). However, from a population dynamics perspective, there is a clear mathematical difference between vector and non-vector multi-host systems: host species contribute either multiplicatively or additively to the pathogen's basic reproductive ratio. This suggests that the ‘sequential’ definition (#7) is most appropriate in this context, although that would also mean including intermediate hosts (e.g. snails for schistosome parasites) as vectors; hence a more appropriate population dynamics definition may be the intersection of the sequential (#7) and micropredator (#3) definitions (figure 1*c*). From an epidemiology and control perspective, it is important to clearly define what a vector is and why that is important before attempting activities such as vector incrimination. The criteria most used for this are those of Barnett [87], which are based on the haematophagous arthropod definition, and may need to be modified or extended if, for example, mobility and sequential transmission are considered to be key criteria.

Having a clear definition of a vector is also important from an evolutionary perspective. As we show above, the sequential feeding aspect of vector transmission is clearly a key point, resulting in different pathogen evolutionary dynamics from those seen under more general multi-host models [41]. It is likely to also be important to consider the extent to which selective pressures on pathogen and vector align; although they may coincide to a degree (and certainly more so than selective pressures acting on trophically transmitted parasites and their intermediate hosts), it is apparent that the selection pressures acting on vectors and vector-borne pathogens do not completely coincide; many pathogens have significant effects on the behaviour [32,33] or survival [88] of vectors. One immediate implication of this is that it is clear that a morbidity-based definition of vectors is overly restrictive. More broadly, it implies that the theoretical frameworks needed to describe vector–pathogen (co-)evolutionary dynamics differ from those needed for pathogen–intermediate host dynamics. Also from the evolutionary perspective, ‘vector shifts’ between insect- and tick-borne transmission occur with some frequency, and this is probably facilitated by similar feeding mode and internal environments (from the perspective of the pathogens or parasites) such as antiviral responses, whereas the mobility of a putative vector is far less important. A useful definition from the evolutionary perspective should therefore reflect this.

More generally, given the plurality of definitions in regular use, we suggest that authors writing about ‘vector-borne diseases’ give careful consideration to whether defining a vector within their system of interest is more likely to help or hinder understanding, and that wherever the term is used the authors clearly define it, and ideally justify the definition chosen. To paraphrase Box's [89] famous comment about modelling: all vector definitions are wrong, but some are (we hope) useful.
